# Study of Seed Ageing in *lpa1-1* Maize Mutant and Two Possible Approaches to Restore Seed Germination

**DOI:** 10.3390/ijms24010732

**Published:** 2023-01-01

**Authors:** Federico Colombo, Andrea Pagano, Stefano Sangiorgio, Anca Macovei, Alma Balestrazzi, Fabrizio Araniti, Roberto Pilu

**Affiliations:** 1 Department of Agricultural and Environmental Sciences—Production, Landscape, Agroenergy, Università degli Studi di Milano, Via G. Celoria 2, 20133 Milan, Italy; 2 Department of Biology and Biotechnology ‘L. Spallanzani’, University of Pavia, Via Ferrata 9, 27100 Pavia, Italy

**Keywords:** phosphorus, *low phytic acid* mutants, plant breeding, aging, seed quality, seed priming, hydropriming, untargeted-metabolomic, environmental sustainability, antioxidant

## Abstract

Phytic acid (PA) is a strong anti-nutritional factor with a key antioxidant role in countering reactive oxygen species. Despite the potential benefits of *low phytic acid* (*lpa*) mutants, the reduction of PA causes pleiotropic effects, e.g., reduced seed germination and viability loss related to seed ageing. The current study evaluated a historical series of naturally aged seeds and showed that *lpa1-1* seeds aged faster as compared to wildtype. To mimic natural ageing, the present study set up accelerated ageing treatments at different temperatures. It was found that incubating the seeds at 57 °C for 24 h, the wildtype germinated at 82.4% and *lpa1-1* at 40%. The current study also hypothesized two possible solutions to overcome these problems: (1) Classical breeding was used to constitute synthetic populations carrying the *lpa1-1* mutation, with genes pushing anthocyanin accumulation in the embryo (R-navajo allele). The outcome showed that the presence of R-navajo in the *lpa1-1* genotype was not able to improve the germinability (−20%), but this approach could be useful to improve the germinability in non-mutant genotypes (+17%). (2) In addition, hydropriming was tested on *lpa1-1* and wildtype seeds, and germination was improved by 20% in *lpa1-1*, suggesting a positive role of seed priming in restoring germination. Moreover, the data highlighted metabolic differences in the metabolome before and after hydropriming treatment, suggesting that the differences in germination could also be mediated by differences in the metabolic composition induced by the mutation.

## 1. Introduction

Iron (Fe) and zinc (Zn) deficiencies are a major challenge in human nutrition, particularly in those countries where a daily cereal diet is based on one single staple crop such as rice, wheat, or maize, etc. The lack of these and other essential minerals in the diet can adversely affect the metabolism [1,2]. Different approaches to increase Fe and Zn levels and improve seed nutritional quality in crops have been proposed, including lowering phytic acid (PA) (myo-inositol-1,2,3,4,5,6-hexakisphosphate, InsP6) in seeds. PA represents the main phosphorus (P) storage form in the seed and is one of the major constraints to micronutrient bioavailability [3]. In fact, due to its negative charge at the physiological pH, PA tends to chelate positively charged cations (such as Fe^2+^, Zn^2+^, K^+^, Mg^2+^, and Ca^2+^), forming phytate mixed salts which accumulate in protein storage vacuoles [4]. During germination, phytate salts are degraded by the activity of the phytase enzyme, releasing free phosphorus and minerals essential for seedlings’ growth [5]. These salts are largely excreted by non-ruminant animals (including humans) as the phytase activity in their digestive system is absent or limited: only 10% of phytate in animal feed is assimilated, while the remainder is excreted, thus contributing to P pollution and water eutrophication [1].

Despite being considered an antinutrient because of these environmental problems and for its chelating effect on minerals, PA plays a key role as an antioxidant compound: by chelating free iron cations, PA is an inhibitor of the iron-driven formation of reactive oxygen species (ROS) and of lipid peroxidation in vitro, thus preserving the viability of seeds [6,7,8]. The prevention of oxidative events is an important antioxidant function of PA within plant seeds. It can help to explain why seeds belonging to many plant species are viable for a long time, even if they contain a potentially dangerous mixture of iron, oxygen, and unsaturated fatty acids [7,8].

In recent decades, mutations that significantly reduce the levels of PA in the seed have been isolated in the major crops, such as maize [9,10,11,12], barley [13,14,15], rice [16,17], wheat [18], soybean [19,20,21], and common bean [22,23]. The development of *low phytic acid* (*lpa*) crops is considered an important goal for nutritional quality improvement [24]. 

In maize, among the three *lpa* mutations isolated so far (*lpa1*, *lpa2*, and *lpa3*), *lpa1* showed the lowest PA content in the seed, leading to a proportional increase in free phosphate, without altering the total P content [9,10]. In the initial screen, 25 *lpa1* alleles were isolated, as reported by Raboy et al. (2001) and Raboy (2009), but many of them were lethal as homozygotes [1,25]. Transposon mutagenesis experiments demonstrated that the *lpa1* gene encodes a multidrug-associated protein (MRP) named *ZmMRP4* (accession number EF586878) [26]. MRP proteins are transmembrane transporters involved in different functions in plants, such as organic ions’ transport, xenobiotic detoxification, transpiration control, and oxidative stress tolerance [27,28]. Most *low phytic acid* mutants carry mutations in genes that code for MRP proteins, resulting in a lack of transfer of PA from the cytosol to the vacuole. 

In the case of maize, four *lpa1* mutants have been isolated and characterized: *lpa1-1* [26], *lpa1-241* [29,30], *lpa1-7* [11], and *lpa1-5525* (not thoroughly characterized) [12]. *lpa1-241* and *lpa1-7* mutants are characterized by high reductions of PA (up to 80–90%), and therefore are not viable in the homozygous state. *lpa1-1* is characterized by a moderate reduction of PA (about 66%) and is the most promising *lpa1* mutant [9]. Unfortunately, the reduction of PA in *lpa1* mutations is associated with various adverse pleiotropic effects on the seed and, in general, on plant performance [31]. Among these pleiotropic effects, reduced seed germination in the field and viability impairment related to seed ageing were previously reported in *lpa* mutants in maize [32], but no experimental data on natural ageing were presented.

Seed ageing is one of the major issues in the storage of seeds. It is defined as deteriorative changes occurring in the seed over time, increasing its vulnerability to external challenges and decreasing its survival ability. The loss of seed viability greatly depends on environmental factors, mainly seed moisture, storage temperature, and relative humidity (RH) [33,34]. The process of seed ageing is normally accompanied by an accumulation of ROS, high concentrations of H_2_O_2_, malondialdehyde, and end-products of lipid peroxidation [35,36,37]. An accelerated ageing test was initially developed to estimate the longevity of seeds in commercial storage [38]. A few years later, the test was evaluated as an indicator of seed vigor in many crops [39] and was successfully related to field emergence [40].

Based on these findings, the present study showed that *lpa1-1* seeds aged faster under room conditions than their related wildtype seeds, leading to a progressive decrease in seed germination and a lower tolerance to adverse storage conditions. The current study also proposed two possible solutions to overcome the problems related to seed germination: (i) First, conventional breeding was used to constitute synthetic maize populations carrying the *lpa1-1* mutation together with regulatory genes pushing the anthocyanin accumulation in the embryo. The authors hypothesized that in this way, it might be possible to compensate for the loss of antioxidant activity caused by PA reduction in *lpa* mutants. (ii) The second approach was based on seed priming technology, a pre-sowing treatment which leads to a physiological state that enables the seed to germinate more efficiently. In both cases, the goal is to find a solution to these pleiotropic effects in the *lpa1-1* mutant, thus improving seed germination and the consequent seedling growth.

Simultaneously, a GC-MS approach was used to profile and explore the metabolic profile of *lpa1-1*-germinating seeds and their control subjected to hydropriming treatment. In fact, although the positive effect of seed priming on seedling emergence and growth is well-reported on different crops, the current study investigated the effect of hydropriming on the metabolic status of *lpa1-1* maize seeds during germination.

## 2. Results

### 2.1. Accelerated Ageing Mimics the Natural Ageing

**Figure 1:**
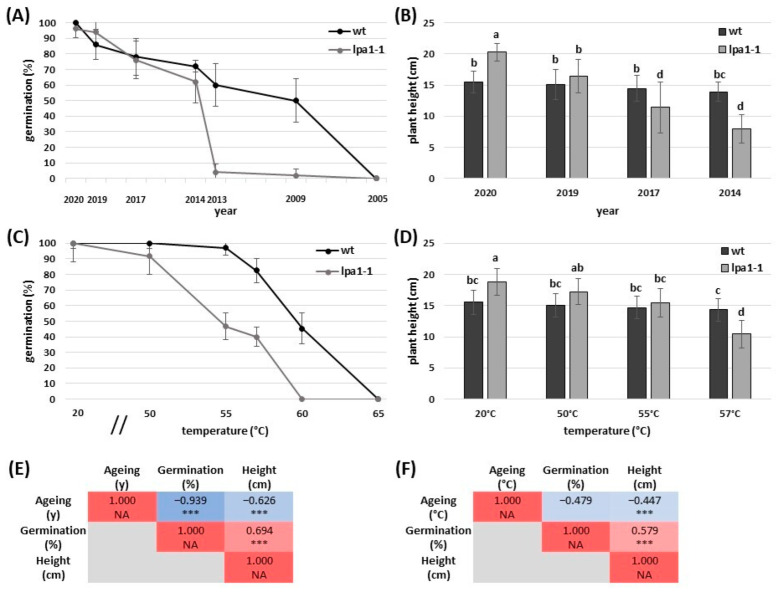
Germination percentage of B73 and *lpa1-1* seeds (**A**) and relative seedlings’ height measured at 13 DAS (**B**). Accelerated seed ageing was performed for 24 hours at 50, 55, 57, 60, and 65 °C with 100% RH (**C**) and plant height was measured at 13 DAS (**D**). Different letters indicate statistically significant differences (Tukey’s test, *p* < 0.05). Heatmap of the correlation analysis performed between years of natural ageing, germination percentage, and height of the plants (**E**). Heatmap of the correlation analysis performed between degrees Celsius (°C) applied for accelerated ageing, germination percentage, and height of the plants (**F**). Pearson’s correlation coefficients are reported. Asterisks indicate statistically significant correlations (*** *p* < 0.001).

In this work, we used seeds of the B73 inbred line and its related *lpa1-1* mutant to study the effect of natural ageing on seed germinability. The experiment was carried out in September 2021, so the seeds harvested in September 2020 were aged by one year, those of September 2019 by two, etc. Seeds of the two genotypes, which had been stored at room temperature (20–25 °C) for several years, were germinated in controlled conditions on germination trays filled with soil, and the germination percentage (%) was noted after 7 days (Figure 1A). It emerged that the mutant was more susceptible to natural seed ageing compared to the wildtype. In fact, if from the first (2020) sampling point the germination of the two genotypes was statistically identical, the trend changed with the seeds of 2013, where the wildtype germinated at 60% while the mutant dropped to 4%. Good germination percentages of the inbred line B73 were also recorded with the seeds of 2009, in which half of the seeds sown were able to germinate (50%) (Figure 1A). Although the germination in controlled conditions remained high in the first years of natural ageing, the vigor and development of the seedlings in the early stages of growth changed. To highlight these differences, the heights of 10 randomly selected plants of each genotype were measured at 13 days after sowing (DAS) (Figure 1B). It emerged that the height of the inbred line B73 remained relatively stable despite the natural ageing (15.44 ± 1.76 cm in 2020 vs. 13.93 ± 1.52 cm in 2014), but the opposite occurred in *lpa1-1*, which lost much of its post-germinative vigor over the years: the seedlings of 2020 reached 20.27 cm, a higher value compared to the wildtype. In fact, in the mutant, the phosphate and the other micronutrients are not chelated and therefore are more bioavailable in the first stages after germination than B73. However, from only one year more of storage, the mutant plants measured 4 cm less than in 2020, while the same parameter dropped to 11.41 cm for 2017 and 7.93 cm for 2014 storage (Figure 1B).

An accelerated ageing treatment was carried out on seeds of both genotypes to mimic natural ageing. After some preliminary tests, a 24 h heat treatment at different temperatures (50, 55, 57, 60 °C) and 100% RH was chosen. A germination test followed the treatment in controlled conditions: the graph clearly shows that the inbred line continued to have a high germination rate despite the heat treatment at 55 °C, while the germination dropped significantly in *lpa1-1* (96.9 vs. 46.6%, respectively). The same trend was maintained at 57 °C (82.4 vs. 40%), while at 60 °C the mutant could no longer germinate (Figure 1C). This parameter was statistically similar for plant height in the inbred line between 20 and 55 °C. In our mutant, the trend was similar to that seen previously in Figure 1B: at room temperature, the height reached 18.74 cm, while it dropped to 11.1 cm with a treatment at 57 °C (Figure 1D).

The correlation analysis between years of natural ageing, germination percentage, and height of the plants highlighted the significant negative correlations between years of ageing with both germination percentage and plant height, as well as a significant positive correlation between germination percentage and plant height (r = +0.694; *p* < 0.001) (Figure 1E). The correlation analysis performed between degrees Celsius (°C) applied for the accelerated ageing, germination percentage, and height of the plants revealed a significant negative correlation between the temperature used for artificial ageing and plant height (r = −0.447; *p* < 0.001), and a significant positive correlation between germination percentage and plant height (r = +0.579; *p* < 0.001) (Figure 1F). Overall, the correlation patterns observed for natural and artificial ageing displayed similar trends.

### 2.2. Genetic Approach to Counteract Seed Ageing

PA plays an important antioxidant function within plant seeds and the defects that characterize *lpa* mutants may be attributable to an anomalous quantity of free iron cations in mutant seeds and to the consequent high level of ROS. A possible approach to defend plant cells from ROS consists in scavenging these toxic free radicals using molecules with antioxidant properties accumulated in the embryo: among them, anthocyanins exhibit a strong effect in counteracting ROS formation. In particular, the R-navajo allele can drive the biosynthesis of anthocyanins in the embryo, where PA is stored. In this work, we compared four synthetic populations that differed only in the presence of the *lpa1-1* and R-nj alleles in homozygous status. The seeds of each genotype were subjected to accelerated ageing (57 °C, 24 h, 100% RH) and were compared to the relative unaged control. The plots were arranged randomly on the germination tray, and three independent replicates were set up. In parallel, five unaged seeds for each genotype were weighed (before and after heat treatment) to determine the seed moisture. These data suggested that seed moisture was statistically identical between the four synthetic populations: Colorless and non-mutant seeds had 7.41% ± 0.33% of water, while the relative colored and non-mutant seeds had 7.26% ± 0.19%. Colorless seeds carrying the *lpa1-1* mutation had 7.47% ± 0.19% of water, and colored mutants had 7.40% ± 0.19%.

**Figure 2:**
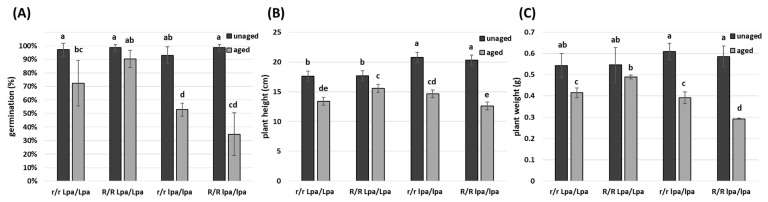
Comparison among the four synthetic populations that differ for the presence of *lpa1-1* and the R-nj allele. Samples subjected to accelerated ageing (57 °C, 24 h) were compared to the respective unaged control. Three independent replicates were performed. Germination percentage was noted at 7 days (**A**), and plant height (**B**) and plant weight (**C**) were measured at 13 days. Different letters indicate statistically significant differences (Tukey’s test, *p* < 0.05).

Aged and unaged seeds were germinated on germination trays, and the germination percentage was noted after 7 days (Figure 2A). As reported in Figure 2A, the accelerated ageing treatment had a different effect on the four genotypes. Considering only the *lpa* material (R/R lpa/lpa vs. r/r lpa/lpa), the germinability dropped by 60% in colored seeds and by 40% in colorless seeds, suggesting that the presence of R-nj allele was not able to improve germinability (−20%). On the other hand, considering the wild genotypes (R/R Lpa/Lpa vs. r/r Lpa/Lpa), the germinability dropped by 8% in colored seeds and by 25% in colorless seeds: the presence of the R-nj allele in this background improved germinability by 17% (Figure 2A).

The same trend was recorded by measuring plant height and weight after 13 days: after the ageing treatment at 57 °C, in R/R Lpa/Lpa the average height of the seedlings decreased only by 12% and the weight by 10%; on the other hand, in colored and mutant seeds (R/R lpa/lpa), the same parameters dropped by 38% and 50%, respectively (Figure 2B), suggesting a possible interference between the *lpa* mutation and anthocyanin accumulation (discussed below).

### 2.3. Hydropriming Improves Germination in Aged Seeds

**Figure 3:**
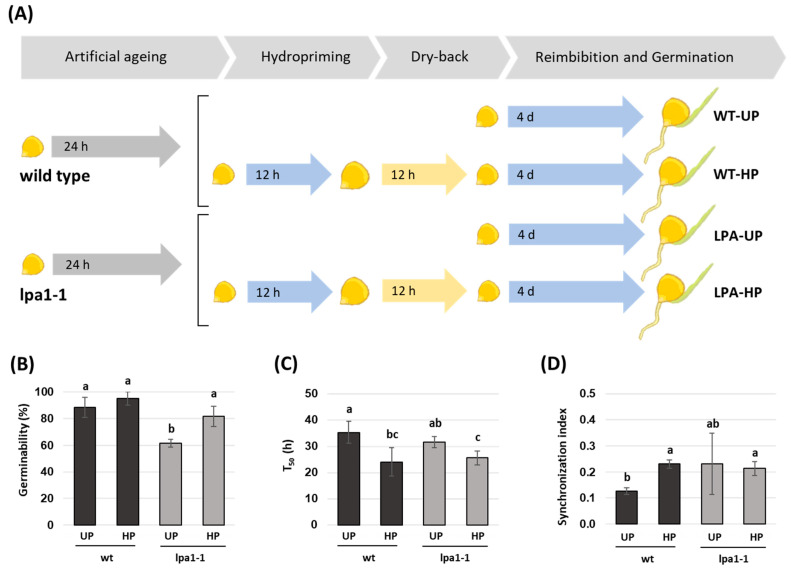
Effects of 12 h-hydropriming on wildtype and *lpa1-1 Zea mays* seeds subjected to accelerated ageing. Overview of the experimental system to compare the effects of hydropriming on wildtype and *lpa1-1 Zea mays* seeds after artificial ageing (**A**). Steps including artificial ageing are indicated with gray arrows, steps involving imbibition are indicated with blue arrows, and dry-back steps are indicated with yellow arrows. Germinability (**B**). T_50_ (**C**). Synchronization index (**D**). UP, unprimed seeds; HP, hydroprimed seeds. Different letters indicate statistically significant differences (Tukey’s test, *p* < 0.05) as analyzed with two-way ANOVA and Tukey’s tests.

The second approach proposed here is based on seed priming technology to tackle seed ageing and improve seed germination in the *lpa1-1* mutant. Based on a literature review, the duration of hydropriming was established at 12 h [41]. An experimental system was established, as detailed in Figure 3A, to assess the effects of 12 h-hydropriming on germination performances in artificially aged wildtype and *lpa1-1* seeds. The beneficial effects of hydropriming compared with unprimed controls were highlighted using three different germination parameters. In unprimed seeds, as expected from previous results (Figure 3B), a decreased germinability was observed in artificially aged *lpa1-1* seeds compared to wildtype seeds (26.67% decrease, Figure 3B), whereas hydropriming significantly improved germinability in artificially aged *lpa1-1* seeds (20.00% increase, Figure 3B). Germination speed, expressed as T_50_, significantly improved in artificially aged wildtype and *lpa1-1* seeds in response to hydropriming, compared to their respective unprimed controls, with an average decrease of 11.33 and 6.02 h in germination time, respectively (Figure 3C). Finally, a significant increase in germination consistency in response to hydropriming, expressed as a higher synchronization index, was observed in artificially aged wildtype seeds, compared with their unprimed controls (81.23% increase, Figure 3D).

### 2.4. Untargeted Metabolomic Analysis

The GC-MS-driven untargeted metabolomic analysis allowed to isolate 324 unknown and 258 putatively annotated compounds. After peak peaking, curation, and discard of incorrect annotations, a list of 158 annotations with a high match factor, total spectra similarity, and RI similarity was reported. The processed data from MS-DIAL with all the analytical and statistical information (retention times, quant mass, signal/noise (S/N), EI spectrum, relative abundances, and univariate and multivariate analysis) are provided in Supplementary Table S1.

**Figure 4:**
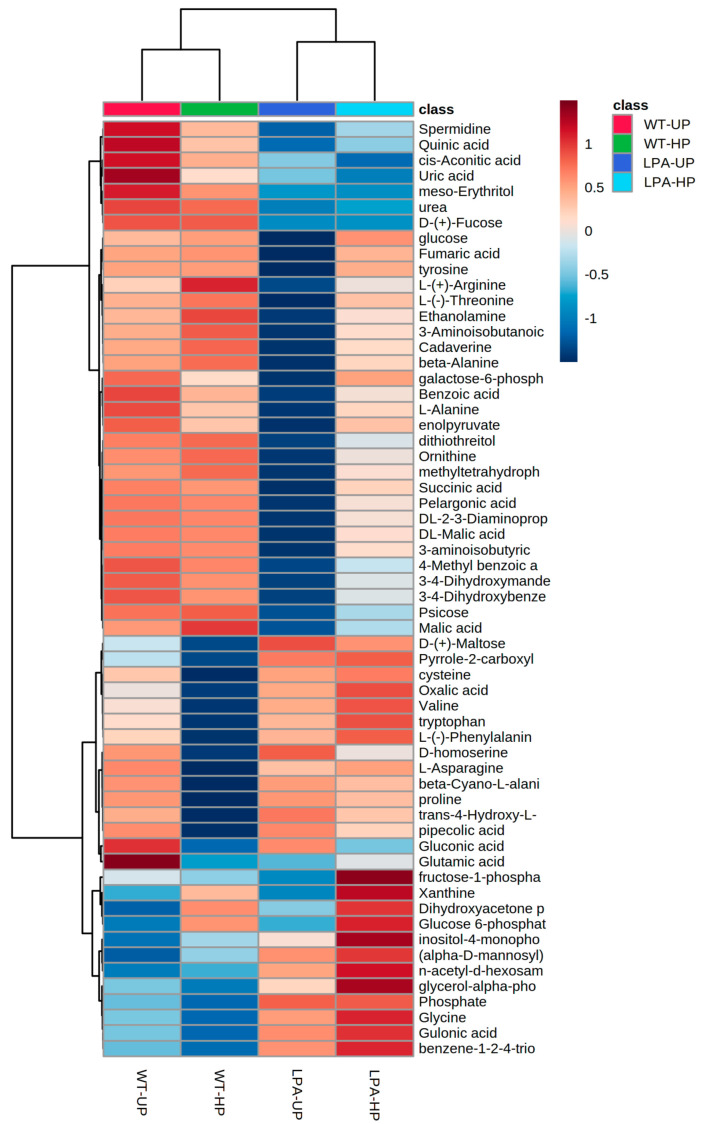
The clustering result is shown as a heatmap (distance measure using Euclidean, and clustering algorithm using Ward D) reporting the top 60 out of 102 metabolites resulting from the ANOVA (LSD test with *p ≤* 0.05 and FDR ≤ 0.05), differentially produced in *Zea mays* seeds of two different genotypes subjected to hydropriming treatment (WT-HP and LPA-HP) and their controls (WT-UP and LPA-UP). Each square represents the different light treatment’s effect on every metabolite’s relative abundance using a false-color scale. Dark red and dark blue colors indicate the relative metabolite abundances, increased and decreased, respectively (*n* = 5).

The univariate ANOVA pointed out that 102 out of 148 metabolites, mainly belonging to chemical classes of the amino acids, organic acids, polyamines, sugars, and sugar alcohols, were significantly altered among the two genotypes and the hydropriming treatment. The top 60 metabolites that resulted significantly from the ANOVA were reported as a heatmap (Figure 4), whereas the complete list of the affected metabolites is reported in Supplementary Table S1. In particular, the metabolic profile was completely different among the two genotypes, and many metabolites (spermidine, urea, L-(+)-Arginine, L-(−)-Threonine) were more abundant in the wildtype, while others in the mutant (valine, glucose-6-phosphate, inositol-4-monophosphate).

**Figure 5:**
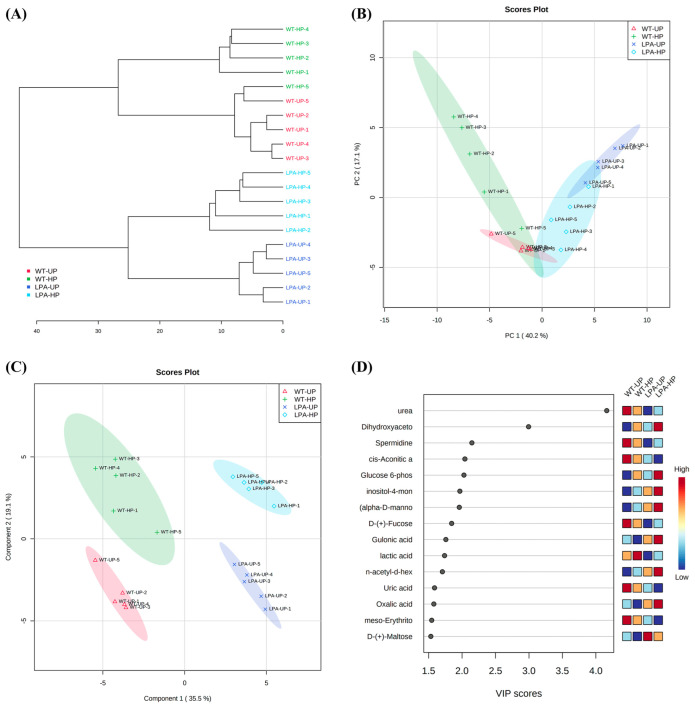
Clustering and multivariate analysis were carried out on *Zea mays* seeds of two different genotypes subjected to hydropriming (WT-HP and LPA-HP) and their unprimed controls (WT-UP and LPA-UP). (**A**) Hierarchical clustering analysis highlighting the result as a dendrogram (distance measure using Euclidean, and clustering algorithm using Ward). (**B**) Scores plot between the selected PCs. The explained variances are shown in brackets. (**C**) Scores plot between the selected PCs. The explained variances are shown in brackets. (**D**) Important features identified by PLS-DA. The colored boxes on the right indicate the relative concentrations of the corresponding metabolite in each group under study (*n* = 5).

The cluster analysis at a lower level pointed out a complete separation among the four groups under evaluation (Figure 5A), suggesting metabolomic differences among genotypes and treatments. In turn, at a higher level, the dendrogram discriminated between two major groups, where each group consisted of unprimed and hydroprimed seeds of a specific genotype, suggesting that the metabolic differences between the two genotypes were the main separation factors (Figure 5A).

Then, data were analyzed through unsupervised multivariate analysis to reduce the dimensionality of the data and visualize samples’ grouping. The PCA’s score plot, built by virtue of the two components PC1 vs. PC2, described 57.3% of the total variability and revealed good discrimination between the samples (Figure 5B). In particular, the highest separations were observed between the unprimed seeds of the two genotypes, located on two separated quadrants (Figure 5B). On the contrary, the hydropriming treatment increased the variability among the replicates, highlighting a separation from their respective unprimed seeds and a weak overlapping, mainly due to samples’ variability (Figure 5B). The loading plots of the PCA pointed out that PC1 was mainly dominated by D-(+)-Maltose, oxalic acid, glycolic acid, spermidine, ornithine, lactic acid, and urea, whereas the PC2 by aspartic acid, glutamic acid, amino-2-propanol, L-asparagine, N-methylalanine, spermidine, proline, and cysteine (Supplementary Table S1).

After PCA, the data were analyzed through the supervised PLS-DA method that, compared to PCA, could classify the observations into groups, giving the most significant predicted indicator variable. As shown in Figure 5C, a complete and clear separation was observed among the four groups under evaluation, and the model was characterized by an R2, Q2, and accuracy higher than 0.9 (Supplementary Table S1), indicating high fitting and good predictivity of the generated model, and significant metabolic change among unprimed and primed samples. The model was built by virtue of the two latent variables (Components 1 and 2), which explained 54.6% of the total variance (Figure 5C). The most significantly altered metabolites contributing to group separation were extracted from the line plots of the X-loadings of the first component of the PLS-DA pairwise comparison models, considering only the variable importance in the projection (VIP) values greater than 1.4 (Figure 5D). The changed metabolites were mainly amino acids, organic acids, and sugars (Figure 5D). Among them, many metabolites increased after the hydropriming treatment: ornithine and lactic acid were higher in the wildtype; vice versa, *lpa1-1* hydroprimed seeds presented higher values of glucose-6-phosphate and inositol-4-monophosphate (Figure 5D).

**Figure 6:**
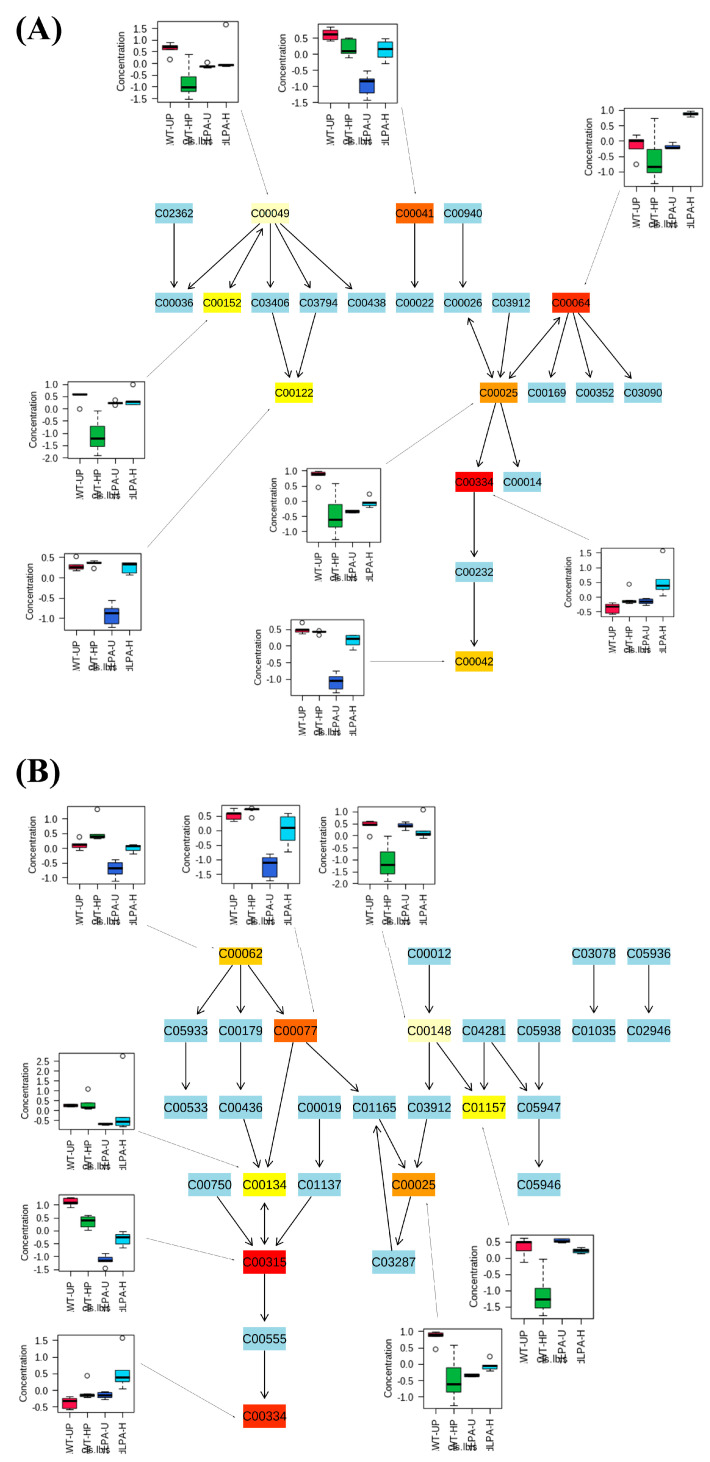
Top two most affected pathways resulted from the pathway analysis carried out with MetPa on *Z. mays* (WT and LPA mutant) unprimed and hydroprimed seeds. (**A**) Alanine aspartate and glutamate metabolism. (**B**) Arginine and proline metabolism. KEGG codes: **C00025**—L-Glutamic acid; **C00041**—L-Alanine; **C00042**—Succinic acid; **C00049**—L-Aspartic acid; **C00062**—L-Arginine; **C00064**—L-Glutamine; **C00077**—Ornithine; **C00122**—Fumaric acid; **C00134**—Putrescine; **C00148**—Proline; **C00152**—L-Asparagine; **C00315**—Spermidine; **C00334**—GABA; **C01157**—trans-4-Hydroxy-L-proline. Light-blue boxes represent metabolites belonging to the pathway that has not been identified during the analysis. Colored boxes from light yellow to red represent identified metabolites: the more the color tends to red, the more significant the data. Data were analyzed through one-way ANOVA (LSD test with *p* ≤ 0.05 and FDR ≤ 0.05). *n* = 5.

The pathway analysis, which combines enrichment and topology analysis, was carried out by comparing the effects of mutation and hydropriming techniques on plant metabolism. The results indicated that 14 pathways, with an impact > 0.2, were significantly affected (Table 1). In particular, the results highlight that the pathways belonging to the amino acid metabolism were the most affected (Table 1). Figure 6 reported the two most affected metabolic routes (alanine, aspartate, glutamate, and arginine and proline metabolism). It should be noted that several metabolites belonging to the classes of amino acids (L-alanine and arginine), organic acids (succinic acid and fumaric acid), and polyamines (ornithine and spermidine) were significantly lower in LPA seeds and then accumulated, reaching almost the control level after the hydropriming treatment (Figure 6A,B). In addition, compounds such as glutamine and GABA, similar to control levels in unprimed LPA seeds, strongly accumulated after the hydropriming treatment (Figure 6A,B). The complete list of metabolites and pathways affected can be found in Supplementary Table S1.

## 3. Discussion

The bioavailability of essential minerals could be a critical factor in the human diet, as their absorption from plant foods is often deficient. One of the major constraints to micronutrient bioavailability is PA, a strong anti-nutritional factor that binds positively charged cations, forming phytate mixed salts [3]. In this context, *low phytic acid* mutants have been isolated in all major crops and are characterized by a reduced amount of PA, followed by a proportional increase in bioavailable P [9,10]. Despite the potential benefits of these mutants, the reduction of PA causes a series of negative pleiotropic effects on the seed and, in general, on plant performance [31]. Among the different *lpa1* mutants in maize, the current study used *lpa1-1* as it is the most promising and compared it with the inbred line B73.

The present study showed that *lpa1-1* seeds aged faster compared to the wildtype, thus causing a progressive decrease in seed germination and a lower tolerance to adverse storage conditions. This study also explored the metabolic changes that occur in *lpa1-1* seeds during germination: a GC-based approach was previously applied to study the metabolic profiles of rice and soybean *lpa* mutants [42,43].

A natural ageing curve was initially constructed to study seed ageing using the seeds of the two genotypes, which had been stored at room temperature in our department (Figure 1A). It emerged that seed germination decreased over the years, but this parameter dropped to 4% in 2013 only in the *lpa1-1* genotype. Although germination remained stable in the two genotypes in the first years of ageing, the mutant seedlings lost vigor much faster than B73 (Figure 1B). The faster development of *lpa1-1* in the early stages was reported in previous studies: due to the PA reduction, minerals were more bioavailable in *lpa* mutants, while they remained chelated in the wildtype [24,44].

Although in the first years of natural ageing the germination of *lpa1-1* remained comparable to the wildtype under controlled conditions, a recent work reported that (unaged) mutant seeds struggle to germinate in open-field conditions, thus impairing the yield [45].

After studying natural ageing, the present study set up an accelerated ageing treatment that allowed us to study seed ageing without having to wait several years. The accelerated ageing test exposed seeds for a short time to two environmental variables, high temperature and high relative humidity, which cause rapid seed deterioration. High-vigor seed lots will withstand these stressful conditions, deteriorate at a slower rate, and have high germination after ageing compared to low-vigor seed lots [46]. Compared to the standard ageing protocols used to evaluate the characteristics of a seed lot, the current study selected higher temperatures (from 50 to 65 °C) and shorter exposition times (24 h) to maximize the differences in seed germination between the two genotypes. By incubating the seeds at 57 °C for 24 h, the inbred germinated at 82.4% and *lpa1-1* at 40%, while when the treatment temperature was 60 °C, only the mutant was no longer able to germinate (Figure 1C).

Considering the relevance of artificial ageing approaches to investigate the effects of natural ageing and long-term storage [47], the correlation analyses displayed in Figure 1E allowed a targeted comparison between natural and artificial ageing evaluated in this study. The correlation patterns observed for natural and artificial ageing with germination percentage and plant height (Figure 1E,F) displayed similar trends, with positive correlations between germination percentage and plant height and negative correlation of ageing (quantified in years for natural ageing and in degrees Celsius for accelerated ageing) with germination percentage and plant height.

Overall, in the first years of storage at room temperature, the germination of the mutant remained comparable to the wildtype, representing a good starting point for the use of the *low phytic acid* trait in crop improvement.

To tackle the limitations related to seed ageing, and in general the reduced seed germination in the mutant, the present study proposed two possible solutions: First, the use of conventional breeding to constitute inbred lines carrying the *lpa1-1* mutation together with genes pushing the anthocyanin accumulation in the embryo. In fact, despite being considered an anti-nutritional factor, PA is a good candidate for protecting the embryo from oxidative processes. For this reason, *lpa* mutants are more exposed to the iron-driven formation of ROS because of their reduced PA content. The present study constituted four synthetic populations that differed only in the presence of the *lpa1-1* and R-nj alleles in homozygous status. The R-navajo allele confers the ability to synthesize and accumulate natural antioxidants in the embryo. The seeds of each genotype were subjected to accelerated ageing (a 24 h treatment at 57 °C was chosen) and were compared to the relatively unaged control. We initially hypothesized that the presence of anthocyanins (R/R lpa/lpa and R/R Lpa/Lpa genotypes) maintained high levels of germination after accelerated ageing thanks to the high antioxidant activity of these pigments [48].

Considering only the *lpa* material (R/R lpa/lpa vs. r/r lpa/lpa), the germinability dropped by 60% in colored seeds and by 40% in colorless seeds, suggesting that the presence of the R-nj allele was not able to improve germinability (−20%) in the mutant line. On the other hand, considering the wild genotypes (R/R Lpa/Lpa vs. r/r Lpa/Lpa), the germinability dropped by 8% in colored seeds and by 25% in colorless seeds: the presence of the R-nj allele improved germinability by 17% in this genetic background (Figure 2A).

In light of these results, it seemed that the presence of R-navajo in the *lpa1-1* genotype did not improve germinability; indeed, it decreased it.

It is possible to explain these results by considering the biosynthesis and compartmentalization of anthocyanins in maize: anthocyanins are cytoplasmically synthesized and transported in the vacuole by *ZmMRP3* activity; however, *ZmMRP3* does not seem to be the only protein involved [49]. Cerino Badone and coworkers suggested a possible role of *ZmMRP4* in the transport of this pigment; in fact, when anthocyanins are transported into the vacuole, due to the acid pH, they assume the typical red color, while if MRP is not functional, they are not transported and accumulate in the less acidic environment of the cytosol, where they retain the bluish color [50]. This alteration was attributed to a defect in the transport of anthocyanins in the vacuole, suggesting that *ZmMRP4* could have an important role in anthocyanin transport. In this scenario, the low germination of the R/R lpa/lpa genotype was induced by the damages caused by seed ageing and the phytotoxicity of anthocyanins in the cytosol. Therefore, using anthocyanins as antioxidants to overcome seed ageing was not functional in improving germination in *lpa1-1* seeds.

However, the results obtained showed that the genotype R/R Lpa/Lpa was the one that germinated best after ageing (Figure 2A). Therefore, the accumulation of anthocyanins did not have a good effect on *lpa1-1*, but this approach is helpful in improving the germinability in non-mutant genotypes. In particular, using seeds that accumulate natural antioxidants could be a good tool when there is a lack of adequate storage facilities and seeds are more exposed to oxidative damage (e.g., underdeveloped countries). Further studies on different genetic materials are needed to validate this hypothesis.

The second approach used in this work to overcome the reduced seed germination was based on seed priming technology, where a 12 h-hydropriming approach was tested on *lpa1-1* and wildtype seeds subjected to accelerated ageing. The treatment improved germination performances in wildtype and *lpa1-1* seeds in terms of germinability percentage and germination speed (Figure 3). In agreement with these results, the positive effects of seed priming in improving seed germination and alleviating the effects of ageing and storage have long been reported in different crop species and explained in terms of enhanced repair and antioxidant responses [51,52]. These results underline the need to explore PA’s roles in seed metabolism, its implications on seed ageing, and the response to priming technology.

The metabolic differences induced by the mutation itself and the hydropriming treatment have also been investigated in this study through an untargeted metabolomic approach, highlighting several metabolomic differences between the wildtype and the *lpa1-1* mutant (Figure 4, Figure 5 and Figure 6 and Table 1). It emerged that there were 102 metabolites significantly altered among the two genotypes and the hydropriming treatment. The distinct and consistent separations among the four groups under evaluation indicate a strong influence of the mutation and the hydropriming treatment on the metabolic profiles (Figure 5C).

Previous metabolomics studies carried out on *low phytic acid* maize kernels highlighted that the seeds of the mutant, compared to WT, were generally characterized by an increase in inorganic phosphorus content, hypothesizing that the accumulation of P-containing metabolites was associated with the reduction of kernels’ dry weight [53]. In agreement with their results, the *lpa* mutant compared to WT was characterized by a higher content in inorganic phosphate; in addition, an accumulation of several P-containing metabolites involved in PA biosynthesis was also observed. In particular, in the *lpa* mutant, the content of glucose-6-phosphate (metabolite involved in the first step of phytic acid biosynthesis) [54], alpha-glycerophosphoric acid, dihydroxyacetone phosphate (alpha-glycerophosphoric acid is converted to glycerone phosphate by the glycerol-3-phosphate dehydrogenase) [55], and inositol 4-monophosphate was higher than in WT and further increased in response to the hydropriming treatment. Similarly, Desai et al. (2014) reported that low-phytate mutations were characterized by an increased production of inositol-phosphate intermediates, highlighting the importance of the inositol phosphate metabolism in PA production [56]. In our study, the pathway analysis highlighted that the inositol phosphate metabolism was significantly altered either by the mutation or by the hydropriming treatment (Supplementary Table S1). The untargeted approach adopted allowed to identify only 3 out 28 compounds belonging to the pathway, keeping the impact lower than 0.2.

Among the biochemical pathways significantly affected by the priming treatment, the alanine aspartate and glutamate metabolism as well as the arginine and proline metabolism were the most impacted. Alterations of these two amino acid pathways were commonly observed during seed responses under different priming treatments [57,58,59].

It has been reported that natural and artificial-induced stress could delay germination, altering the seed metabolic profile. Ruan and coworkers [60] reported that high-pressure stress applied on barley seeds induced a delay of germination accompanied by a reduction of several metabolites, such as urea, sugars (glucose), amino acids (alanine, aspartate, tyrosine, phenylalanine, and tryptophan), and organic acids (citric acid, malic acid). All those metabolites were significantly reduced in the LPA-UP mutant compared to WT-UP, suggesting that the down-accumulation of metabolites induced by the mutation could interfere with the germination process, delaying and/or inhibiting it. On the contrary, after the HP treatment, several metabolites (i.e., L-alanine, arginine, succinic acid, fumaric acid, ornithine, and spermidine, among others), whose abundance in LPA-UP seeds was lower than the control, up-accumulated, reaching control abundances. These results suggest that the HP treatment allowed several metabolic and biochemical activities, such as the hydrolyzation of the reserve proteins, leading to the production of free amino acids, which were then interconverted and used in the synthesis of new compounds.

Hydropriming treatment, which speeds up the imbibition process, could have activated the catabolic process and the bioavailability of nutrients that were stored in their free form during the seeds’ dehydration and that would become promptly available immediately after sowing, enhancing the germination process, as observed in this study (Figure 3B).

It was observed that during the remobilization of seed reserves, the primary metabolism is activated. After seed imbibition, the content of amino acids, related organic acids, and phosphorylated sugars, such as glucose-6-phosphate and fructose-6-phosphate, constantly increase as a consequence of catabolic processes’ activation. In particular, the phosphorylated sugars are pivotal for energy provision since they are the way by which the sugars enter glycolysis [61]. In our experiments, the glucose-6-phosphate and fructose-6-phosphate content significantly increased in both WT-HP and LPA-HP, and a similar trend was also observed in glucose content. On the contrary, in WT-HP and LPA-HP, an opposite trend was observed on several amino acids (i.e., aspartic acid, lysine, cysteine, and methionine) known to play a pivotal role during germination, which decreased their content in WT-HP and increased in LPA-HP.

In Arabidopsis, the aspartate amino acids family is vital for germination [61]. Similarly, the amino acid lysine, deriving from aspartate metabolism, was proven to be involved in providing substrates for the TCA cycle, which provide the energy necessary for the biochemical processes involved in germination [62]. It should be noted that, especially in LPA-HP seeds, an accumulation of TCA cycles’ intermediates, such as fumaric acid, succinic acid, malic acid, citric acid, and phosphoenolpyruvate, was observed.

Overall, the present study suggested that wildtype and *lpa1-1* present significantly different metabolic signatures before and after priming treatment. Probably, these differences are involved in their different germination vigor and response to seed ageing.

## 4. Materials and Methods

### 4.1. Genetic Material

The *lpa1-1* mutation introgressed in the B73 inbred line was kindly provided by USDA ARS, Aberdeen, ID, USA. The inbred line B73 [63] was provided by the germplasm bank at DISAA, Department of Agricultural and Environmental Sciences—Production Landscape, Agroenergy, University of Milan, Italy. Seeds of B73 and its relative *lpa1-1* mutant were used to study the effect of natural and accelerated ageing on seed germinability.

### 4.2. Ageing Conditions and Germination Tests

The seeds used here had been stored at room temperature (20–25 °C) in airtight plastic containers for several years in the germplasm bank at DISAA, Department of Agricultural and Environmental Sciences—Production Landscape, Agroenergy, University of Milan, Italy.

For each point of the natural ageing curve, 100 seeds of B73 and its relative mutant *lpa1-1* were germinated. To mimic natural ageing, the accelerated ageing involves the exposure of samples of seeds to adverse conditions for a specific period [38]. Accelerated ageing was carried out in a thermostatic oven where the desired conditions were maintained. For each point of the accelerated ageing curve, 100 seeds of B73 and *lpa1-1* were treated for 24 h at different temperatures (50, 55, 57, 60, 65 °C) in sealed glass boxes with 100% RH. An unaged seed sample stored at room temperature (20 °C) was used as a control.

The accelerated ageing treatment was followed by a germination test in controlled conditions to determine the percentage of surviving seeds. For both natural and accelerated ageing, seeds were germinated in a seed germination tray filled with S.Q.10 substrate (peat, sand, compost; Vigorplant) and plants were grown under a long-day photoperiod (16 h light/8 h dark) in a growth chamber with controlled temperature (25 °C night/30 °C day) and with photon fluence of 270 mmol m^−2^ s^−1^. At 7 DAS (days after sowing), the seed germination percentage was noted, and the height of 10 randomly selected seedlings for each sampling point was measured at 13 DAS.

The Pearson’s correlation coefficient and the *p*-values of the correlations were calculated using MetaboAnalyst 5.0 [64] to assess the correlations between ageing (quantified in years for natural ageing and in degrees Celsius for artificial ageing), germination percentage, and height of the plants.

### 4.3. Constitution of the New Genetic Material and Plot Experiment

**Figure 7:**
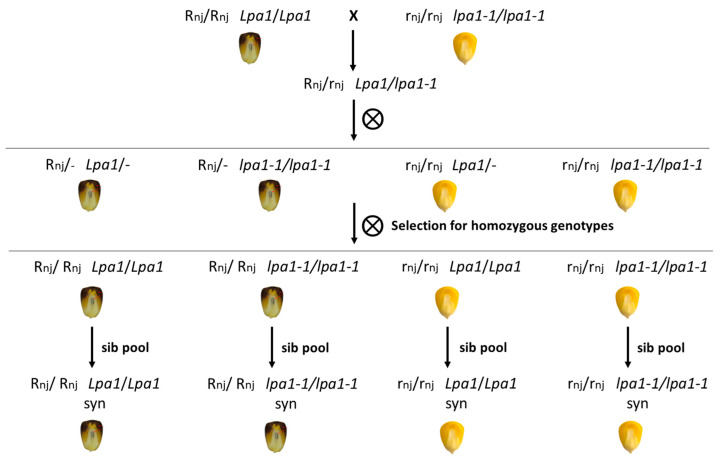
The pedigree scheme used to obtain the four synthetic populations that differ only in the presence of the *lpa1-1* and R-nj alleles in homozygosity. The symbol “-“ means either dominant or recessive alleles. R-nj is the abbreviation of the R-navajo allele.

The present study used conventional breeding to constitute four synthetic populations. The genetic materials here were obtained crossing the mutant *lpa1-1/lpa1-1* (in B73) with the line carrying pigmented seeds, R-navajo (R-nj), provided by the germplasm bank at DISAA, University of Milan, Italy. The R-nj allele can synthesize and accumulate natural antioxidants (anthocyanins) in the embryo, where PA is stored. The F1 obtained was selfed an F2 population scored by Chen’s assay and genotyped for all the possible genotypes in homozygosity. Following three cycles of sib crossing, four synthetic populations differing in the presence of the *lpa1-1* and R-nj in homozygosity were obtained (Figure 7).

In every cycle, the presence of the *low phytic acid* trait in the material selected was confirmed through chemical and molecular methods, according to Colombo et al. [45].

Seeds of the four synthetic populations obtained were subjected to accelerated ageing for 24 h, and a temperature of 57 °C was chosen for the treatment. For each genotype, 3 independent replicates with 25 seeds per replicate were germinated together with the respective unaged control (4 aged, 4 unaged × 3 rep). Seed germination was carried out in the growth chamber, as described in the previous paragraph. At 7 DAS, the seed germination percentage was noted, and at 13 DAS all the seedlings germinated were cut at the base, and the height and weight of these seedlings were measured.

Five representative seeds for each genotype were weighed before and after a heat treatment at 80 °C in the thermostatic oven to calculate seed moisture until a constant weight was achieved.

### 4.4. Hydropriming Protocol and Germination Test

To assess the effects of hydropriming on artificially aged wildtype and *lpa1-1* seeds, accelerated ageing was carried out as previously indicated (57 °C for 24 h). For hydropriming treatments, seeds were transferred in sealed trays (15 × 20 cm) containing a layer of filter paper moistened with 20 mL of distilled water and left soaking for 12 h. After imbibition, hydroprimed seeds were distributed on a layer of absorbing paper and air-dried (dry-back) for 12 h.

For germination tests, artificially aged wildtype and *lpa1-1* seeds subjected to hydropriming (WT-HP and LPA-HP, respectively) and unprimed controls consisting of artificially aged wildtype and *lpa1-1* seeds not subjected to hydropriming (WT-UP and LPA-UP, respectively) were distributed into trays (10 × 15 cm) containing a layer of filter paper moistened with 10 mL of distilled water. Trays were sealed with transparent film to prevent water evaporation. Germination was assessed every 12 h for 4 days, starting from the imbibition of unprimed seeds and the re-imbibition of hydroprimed seeds. Seeds with visible protrusion of the primary root were considered germinated. The following parameters were calculated: G (germinability, final germination percentage), T_50_ (time required to reach 50% of the final germination percentage), and Z (synchronization index) [65]. Three independent replicates with twenty seeds per replicate were analyzed for each treatment. Statistical analyses were carried out using two-way ANOVA and Tukey–Kramer tests as post hoc tests (*p* ≤ 0.05). Statistical analysis was conducted using the software developed by Assaad and colleagues [66].

### 4.5. Metabolomic Analysis

#### 4.5.1. Samples’ Extraction and Derivatization

Maize seeds were collected and powdered to quench the endogenous metabolism. Then, 100 mg of freshly homogenized seed powder was used for each treatment and replicate. Extraction was performed by adding 1400 µL of a cold (−20 °C) methanol:water (1:1) solution acidified with acetic acid (5%). Samples were vortexed for 10 s after adding 60 µL of ribitol (0.2 mg/mL of stock in ddH_2_O) as an internal quantitative standard for the polar phase. Samples were transferred in a thermomixer at 70 °C, shaken for 15 min (950 rpm), and further centrifuged for 10 min at 11,000× *g*. The supernatants were collected and transferred to glass vials, where 750 µL of CHCl_3_ (−20 °C) was added. All the samples were vortexed for 10 s and then centrifuged for another 15 min at 2200× *g*, 150 µL for each treatment, and replicates were collected, transferred to a 1.5 mL tube, and dried in a vacuum concentrator without heating.

After the addition of 40 µL of methoxyamine hydrochloride (20 mg/mL in pyridine), the samples were incubated for 2 h in a Thermomixer (950 rpm) at 37 °C. Methoxyaminated samples were then silylated by adding 70 µL of MSTFA to the aliquots. Samples were further shaken for 30 min at 37 °C. For analysis, derivatized samples (110 µL) were then transferred into glass vials suitable for the GC-MS autosampler [67].

#### 4.5.2. GC-MS Analysis

The derivatized extracts were injected into a 5MS capillary column (30 m × 0.25 mm × 0.25 µm + 10 m of pre-column) using a gas chromatograph apparatus (GC 7890A, Agilent) equipped with a single quadrupole mass spectrometer (MS 5975C, Agilent). The injector and source were set at 230 and 250 °C, respectively. The transfer line temperature was settled to 300 °C, whereas the MS quadrupole to 150 °C. Then, 1 µL of sample was injected in splitless mode with a helium flow of 1 mL/min using the following programmed temperature: isothermal 5 min at 70 °C, followed by a 11 °C/min ramp to 300 °C and a final 5 min heating at 300 °C. Mass spectra were recorded in electronic impact (EI) mode at 70 eV, scanning at a 40–600 *m*/*z* range, with a scan time of 0.2 s. Mass spectrometric solvent delay was settled as 9 min. Pooled samples that served as quality controls (QCs), n-alkane standards (C10–C40, all even), and blank solvents were injected at scheduled intervals for instrumental performance, tentative identification, and monitoring of shifts in retention indices (RI) [67].

#### 4.5.3. MS-DIAL Analysis

Raw chromatograms were analyzed using the open-source software MS-DIAL 4.9, and peak intensity extraction and annotation were carried out as previously reported by Misra et al. [67]. The metabolomics standards initiative (MSI) guidelines for metabolite identification were followed for metabolite annotation and assignment of the EI-MS spectra [68]. Here, metabolites were annotated at Level 2 (identification based on the spectral database using a match factor > 80%).

#### 4.5.4. Statistical Analysis

GC-MS data were statistically analyzed by using MetaboAnalyst version 5.0 [64]. Briefly, relative Lowess-normalized abundance values from the MS-DIAL outputs were Log-transformed and Pareto-scaled before performing univariate (ANOVA), cluster analysis (using Euclidean distance and Ward as the clustering algorithms), and multivariate analysis, i.e., PCA (principal component analysis) and PLS-DA (partial least squares discriminant analysis). The PLS-DA’s Variable Importance of Projection (VIP), with a VIP score ≥ 1.4, was also reported. To avoid overfitting, the PLS-DA model was validated using R2 and Q2 as a performance measure, the 10-fold cross-validation, and setting in the permutation test a permutation number of 20, considered significant with *p* ≤ 0.05 (see figures reported in Supplementary Table S1—PLS-DA loadings). Further, a pathway analysis was performed, using the MetaboAnalyst tool MetPA, to identify and evaluate the effects of the mutation and the priming treatments on germinating seed metabolism. Only pathways with a *p*-value and false discovery rate (FDR) ≤ 0.05 and an impact higher than 0.2 were considered significantly affected. See Supplementary Table S1 for a deeper understanding of the univariate and multivariate approach in metabolomics experiments and for the full list of metabolic pathways affected.

## 5. Conclusions

Seed ageing reduces seed quality, storage duration, and germinability, causing a loss of vigor and making the seeds less viable in the field. The present work highlighted for the first time that *lpa1-1* seeds aged faster than the wildtype and confirmed the suitability of accelerated ageing treatment as an informative approach to highlight the pleiotropic effects associated with a low PA content. Although the introgression R-navajo allele did not yield the desired result with the low PA mutant, the used seeds that accumulated natural antioxidants could be a good tool to improve the germinability in other wildtype material. This approach should consider the introgression of other antioxidant molecules accumulated in the embryo, e.g., carotenoids or tocopherols. The other approach used in this work was seed priming: a pre-imbibition in water (hydropriming) seemed to be a promising solution for restoring germination rates and improving seed viability in *low phytic acid* mutants. Moreover, the metabolic profiles obtained in *lpa1-1* germinating seeds highlighted that the pathways belonging to the amino acid metabolism were the most affected.

Considering the current findings achieved with hydropriming, the way forward for future work should focus on hormonal seed priming, using molecules such as gibberellic acid (GA_3_) to increase the germination of the *lpa1-1* mutant. These experiments should be accompanied by multi-year field evaluations to overcome the reduced seed germination of *lpa1-1* in specific local environments, which remains the major issue for breeders.

## Figures and Tables

**Table 1:** Results from pathway analysis with MetPa carried out on *Z. mays* (WT and LPA mutant) unprimed and hydroprimed seeds.

Metabolites	Total Cmpd	Hits	*p*-Value	FDR	Impact
Alanine aspartate and glutamate metabolism	22	8	1.39 × 10^−8^	3.97 × 10^−7^	0.77698
Arginine and proline metabolism	28	8	4.00 × 10^−7^	2.07 × 10^−6^	0.63167
Tryptophan metabolism	23	2	1.58 × 10^−5^	3.45 × 10^−5^	0.5862
Glycine serine and threonine metabolism	33	6	7.72 × 10^−9^	3.97 × 10^−7^	0.57964
Phenylalanine metabolism	12	1	0.000234	0.000333	0.42308
Starch and sucrose metabolism	22	5	2.41 × 10^−7^	1.53 × 10^−6^	0.4125
Isoquinoline alkaloid biosynthesis	6	2	0.007983	0.009286	0.41176
beta-Alanine metabolism	18	6	1.01 × 10^−6^	4.80 × 10^−6^	0.4008
Cyanoamino acid metabolism	26	6	2.82 × 10^−6^	8.91 × 10^−6^	0.375
Arginine biosynthesis	18	7	1.87 × 10^−6^	6.97 × 10^−6^	0.34079
Tyrosine metabolism	18	3	1.65 × 10^−7^	1.42 × 10^−6^	0.27568
Pantothenate and CoA biosynthesis	23	5	1.92 × 10^−5^	4.05 × 10^−5^	0.24406
Citrate cycle (TCA cycle)	20	5	1.36 × 10^−6^	5.55 × 10^−6^	0.23269
Glyoxylate and dicarboxylate metabolism	29	8	9.20 × 10^−8^	1.42 × 10^−6^	0.20626

Total Cmpd: the total number of compounds in the pathway; Hits: the matched number from the uploaded data; *p*-value: the original *p*-value calculated from the enrichment analysis; FDR: false discovery rate; Impact: the pathway impact value calculated from pathway topology analysis. *n* = 5.

## Data Availability

Not applicable.

## Supplementary Materials

The supporting information can be downloaded at: https://www.mdpi.com/article/10.3390/ijms24010732/s1.

## References

[1] Raboy V., Young K.A., Dorsch J.A., Cook A. (2001). Genetics and breeding of seed phosphorus and phytic acid. J. Plant Physiol..

[2] Schlemmer U., Frølich W., Prieto R.M., Grases F. (2009). Phytate in foods and significance for humans: Food sources, intake, processing, bioavailability, protective role and analysis. Mol. Nutr. Food Res..

[3] Raboy V., Larkins B.A., Asil I.K. (1997). Accumulation and Storage of Phosphate and Minerals.

[4] Raboy V. (2002). Progress in Breeding Low Phytate Crops. Am. Soc. Nutr. Sci..

[5] Laboure A.M., Gagnon J., Lescure A.M. (1993). Purification and characterization of a phytase (myo-inositol-hexakisphosphate phosphohydrolase) accumulated in maize (*Zea mays*) seedlings during germination. Biochem. J..

[6] Graf E., Mahoney J.R., Bryant R.G., Eaton J.W. (1984). Iron-catalyzed hydroxyl radical formation. Stringent requirement for free iron coordination site. J. Biol. Chem..

[7] Graf E., Empson K.L., Eaton J.W. (1987). Phytic acid. A natural antioxidant. J. Biol. Chem..

[8] Graf E., Eaton J.W. (1990). Antioxidant functions of phytic acid. Free Radic. Biol. Med..

[9] Raboy V., Gerbasi P.F., Young K.A., Stoneberg S.D., Pickett S.G., Bauman A.T., Murthy P.P.N., Sheridan W.F., Ertl D.S. (2000). Origin and seed phenotype of maize low phytic acid 1-1 and low phytic acid 2-1. Plant Physiol..

[10] Pilu R., Panzeri D., Gavazzi G., Rasmussen S.K., Consonni G., Nielsen E. (2003). Phenotypic, genetic and molecular characterization of a maize low phytic acid mutant (lpa241). Theor. Appl. Genet..

[11] Cerino Badone F., Amelotti M., Cassani E., Pilu R. (2012). Study of low phytic acid1-7 (lpa1-7), a new ZmMRP4 mutation in maize. J. Hered..

[12] Borlini G., Rovera C., Landoni M., Cassani E., Pilu R. (2019). Lpa1-5525: A new lpa1 mutant isolated in a mutagenized population by a novel non-disrupting screening method. Plants.

[13] Larson S.R., Young K.A., Cook A., Blake T.K., Raboy V. (1998). Linkage mapping of two mutations that reduce phytic acid content of barley grain. Theor. Appl. Genet..

[14] Rasmussen S.K., Hatzack F. (1998). Identification of two low-phytate barley (Hordeum vulgare L.) grain mutants by TLC and genetic analysis. Hereditas.

[15] Bregitzer P., Raboy V. (2006). Effects of four independent low-phytate mutations on barley agronomic performance. Crop Sci..

[16] Larson S.R., Rutger J.N., Young K.A., Raboy V. (2000). Isolation and genetic mapping of a non-lethal rice (*Oryza sativa* L.) low phytic acid 1 mutation. Crop Sci..

[17] Liu Q.L., Xu X.H., Ren X.L., Fu H.W., Wu D.X., Shu Q.Y. (2007). Generation and characterization of low phytic acid germplasm in rice (*Oryza sativa* L.). Theor. Appl. Genet..

[18] Guttieri M., Bowen D., Dorsch J.A., Raboy V., Souza E. (2004). Identification and characterization of a low phytic acid wheat. Crop Sci..

[19] Wilcox J.R., Premachandra G.S., Young K.A., Raboy V. (2000). Isolation of high seed inorganic P, low-phytate soybean mutants. Crop Sci..

[20] Hitz W.D., Carlson T.J., Kerr P.S., Sebastian S.A. (2002). Biochemical and molecular characterization of a mutation that confers a decreased raffinosaccharide and phytic acid phenotype on soybean seeds. Plant Physiol..

[21] Yuan F.J., Zhao H.J., Ren X.L., Zhu S.L., Fu X.J., Shu Q.Y. (2007). Generation and characterization of two novel low phytate mutations in soybean (*Glycine max* L. Merr.). Theor. Appl. Genet..

[22] Campion B., Sparvoli F., Doria E., Tagliabue G., Galasso I., Fileppi M., Bollini R., Nielsen E. (2009). Isolation and characterisation of an lpa (low phytic acid) mutant in common bean (*Phaseolus vulgaris* L.). Theor. Appl. Genet..

[23] Cominelli E., Confalonieri M., Carlessi M., Cortinovis G., Daminati M.G., Porch T.G., Losa A., Sparvoli F. (2018). Phytic acid transport in *Phaseolus vulgaris*: A new low phytic acid mutant in the PvMRP1 gene and study of the PvMRPs promoters in two different plant systems. Plant Sci..

[24] Colombo F., Bertagnon G., Ghidoli M., Pesenti M., Giupponi L., Pilu R. (2022). Low-Phytate Grains to Enhance Phosphorus Sustainability in Agriculture: Chasing Drought Stress in lpa1-1 Mutant. Agronomy.

[25] Raboy V. (2009). Seed Phosphate.

[26] Shi J., Wang H., Schellin K., Li B., Faller M., Stoop J.M., Meeley R.B., Ertl D.S., Ranch J.P., Glassman K. (2007). Embryo-specific silencing of a transporter reduces phytic acid content of maize and soybean seeds. Nat. Biotechnol..

[27] Swarbreck D., Ripoll P.J., Brown D.A., Edwards K.J., Theodoulou F. (2003). Isolation and characterisation of two multidrug resistance associated protein genes from maize. Gene.

[28] Klein M., Burla B., Martinoia E. (2006). The multidrug resistance-associated protein (MRP/ABCC) subfamily of ATP-binding cassette transporters in plants. FEBS Lett..

[29] Pilu R., Landoni M., Cassani E., Doria E., Nielsen E. (2005). The maize lpa241 mutation causes a remarkable variability of expression and some pleiotropic effects. Crop Sci..

[30] Pilu R., Panzeri D., Cassani E., Badone F.C., Landoni M., Nielsen E. (2009). A paramutation phenomenon is involved in the genetics of maize low phytic acid1-241 (lpa1-241) trait. Heredity.

[31] Colombo F., Paolo D., Cominelli E., Sparvoli F., Nielsen E., Pilu R. (2020). MRP Transporters and Low Phytic Acid Mutants in Major Crops: Main Pleiotropic Effects and Future Perspectives. Front. Plant Sci..

[32] Doria E., Galleschi L., Calucci L., Pinzino C., Pilu R., Cassani E., Nielsen E. (2009). Phytic acid prevents oxidative stress in seeds: Evidence from a maize (*Zea mays* L.) low phytic acid mutant. J. Exp. Bot..

[33] Groot S.P.C., Surki A.A., De Vos R.C.H., Kodde J. (2012). Seed storage at elevated partial pressure of oxygen, a fast method for analysing seed ageing under dry conditions. Ann. Bot..

[34] Li L., Wang F., Li X., Peng Y., Zhang H., Hey S., Wang G., Wang J., Gu R. (2019). Comparative analysis of the accelerated aged seed transcriptome profiles of two maize chromosome segment substitution lines. PLoS ONE.

[35] Debeaujon I., Léon-Kloosterziel K.M., Koornneef M. (2000). Influence of the testa on seed dormancy, germination, and longevity in Arabidopsis. Plant Physiol..

[36] Murthy U.M.N., Kumar P.P., Sun W.Q. (2003). Mechanisms of seed ageing under different storage conditions for *Vigna radiata* (L.) Wilczek: Lipid peroxidation, sugar hydrolysis, Maillard reactions and their relationship to glass state transition. J. Exp. Bot..

[37] Xin X., Tian Q., Yin G., Chen X., Zhang J., Ng S., Lu X. (2014). Reduced mitochondrial and ascorbate-glutathione activity after artificial ageing in soybean seed. J. Plant Physiol..

[38] Delouche J.C., Baskin C.C. (1973). Accelerated aging techniques for predicting the relative storability of seed lots. Proceedings.

[39] TeKrony D.J., Ibrahim A.E., TeKrony D.M., Egli D.B. (1993). Accelerated aging techniques for evaluating Sorghum seed vigor. J. Seed Technol..

[40] Woltz J.M., TeKrony D.M. (2001). Accelerated Aging Test for Corn Seed. Seed Technol..

[41] Wattanakulpakin P., Photchanachai S., Miyagawa S., Ratanakhanokchai K. (2012). Loss of maize seed vigor as affected by biochemical changes during hydropriming. Crop Sci..

[42] Frank T., Seumo Meuleye B., Miller A., Shu Q.Y., Engel K.H. (2007). Metabolite profiling of two low phytic acid (lpa) rice mutants. J. Agric. Food Chem..

[43] Frank T., Nörenberg S., Engel K.H. (2009). Metabolite profiling of two novel low phytic acid (lpa) soybean mutants. J. Agric. Food Chem..

[44] Landoni M., Cerino Badone F., Haman N., Schiraldi A., Fessas D., Cesari V., Toschi I., Cremona R., Delogu C., Villa D. (2013). Low phytic acid 1 mutation in maize modifies density, starch properties, cations, and fiber contents in the seed. J. Agric. Food Chem..

[45] Colombo F., Sangiorgio S., Abruzzese A., Bononi M., Tateo F., Singh S.K., Nocito F.F., Pilu R. (2022). The Potential of Low Phytic Acid1-1 Mutant in Maize (*Zea mays* L.): A Sustainable Solution to Non-Renewable Phosphorus. Front. Biosci..

[46] Tekrony D.M. (2005). Accelerated Aging Test: Principles and Procedures. Seed Technol..

[47] Agacka-Mołdoch M., Arif M.A.R., Lohwasser U., Doroszewska T., Qualset C.O., Börner A. (2016). The inheritance of wheat grain longevity: A comparison between induced and natural ageing. J. Appl. Genet..

[48] Petroni K., Pilu R., Tonelli C. (2014). Anthocyanins in corn: A wealth of genes for human health. Planta.

[49] Goodman C.D., Casati P., Walbot V. (2004). A multidrug resistance-associated protein involved in anthocyanin transport in Zea mays. Plant Cell.

[50] Badone F.C., Cassani E., Landoni M., Doria E., Panzeri D., Lago C., Mesiti F., Nielsen E., Pilu R. (2010). The low phytic acid1-241 (lpa1-241) maize mutation alters the accumulation of anthocyanin pigment in the kernel. Planta.

[51] Tilden R.L., West S.H. (1985). Reversal of the effects of aging in soybean seeds. Plant Physiol..

[52] Yan H., Jia S., Mao P. (2020). Melatonin priming alleviates aging-induced germination inhibition by regulating β-oxidation, protein translation, and antioxidant metabolism in oat (*Avena sativa* L.) seeds. Int. J. Mol. Sci..

[53] Hazebroek J., Harp T., Shi J., Wang H. (2007). Metabolomic Analysis of Low Phytic Acid Maize Kernels. Concepts in Plant Metabolomics.

[54] Sharma S., Hari L., Anandkumar D., Tyagi A., Muthumilarasan M. (2022). An insight into phytic acid biosynthesis and its reduction strategies to improve mineral bioavailability. Nucleus.

[55] Shen W., Wei Y., Dauk M., Zheng Z., Zou J. (2003). Identification of a mitochondrial glycerol-3-phosphate dehydrogenase from Arabidopsis thaliana: Evidence for a mitochondrial glycerol-3-phosphate shuttle in plants. FEBS Lett..

[56] Desai M., Rangarajan P., Donahue J.L., Williams S.P., Land E.S., Mandal M.K., Phillippy B.Q., Perera I.Y., Raboy V., Gillaspy G.E. (2014). Two inositol hexakisphosphate kinases drive inositol pyrophosphate synthesis in plants. Plant J..

[57] Zanganeh R., Jamei R., Rahmani F. (2018). Impacts of seed priming with salicylic acid and sodium hydrosulfide on possible metabolic pathway of two amino acids in maize plant under lead stress. Mol. Biol. Res. Commun..

[58] Li Y., Liang L., Li W., Ashraf U., Ma L., Tang X., Pan S., Tian H., Mo Z. (2021). ZnO nanoparticle-based seed priming modulates early growth and enhances physio-biochemical and metabolic profiles of fragrant rice against cadmium toxicity. J. Nanobiotechnol..

[59] Zhou X., Jia X., Zhang Z., Chen K., Wang L., Chen H., Yang Z., Li C., Zhao L. (2022). AgNPs seed priming accelerated germination speed and altered nutritional profile of Chinese cabbage. Sci. Total Environ..

[60] Ruan Y., Cai Z., Deng Y., Pan D., Zhou C., Cao J., Chen X., Xia Q. (2021). An untargeted metabolomic insight into the high-pressure stress effect on the germination of wholegrain *Oryza sativa* L. Food Res. Int..

[61] Fait A., Angelovici R., Less H., Ohad I., Urbanczyk-Wochniak E., Fernie A.R., Galili G. (2006). Arabidopsis seed development and germination is associated with temporally distinct metabolic switches. Plant Physiol..

[62] Angelovici R., Fait A., Fernie A.R., Galili G. (2011). A seed high-lysine trait is negatively associated with the TCA cycle and slows down Arabidopsis seed germination. New Phytol..

[63] Gerdes J.T., Behr C.F., Coors J.G., Tracy W.F. (1993). Compilation of North American Maize Breeding Germplasm.

[64] Pang Z., Chong J., Zhou G., De Lima Morais D.A., Chang L., Barrette M., Gauthier C., Jacques P.É., Li S., Xia J. (2021). MetaboAnalyst 5.0: Narrowing the gap between raw spectra and functional insights. Nucleic Acids Res..

[65] Ranal M.A., De Santana D.G. (2006). How and why to measure the germination process?. Rev. Bras. Bot..

[66] Assaad H.I., Hou Y., Zhou L., Carroll R.J., Wu G. (2015). Rapid publication-ready MS-Word tables for two-way ANOVA. Springerplus.

[67] Misra B.B., Das V., Landi M., Abenavoli M.R., Araniti F. (2020). Short-term effects of the allelochemical umbelliferone on Triticum durum L. metabolism through GC–MS based untargeted metabolomics. Plant Sci..

[68] Sansone S.-A., Fan T., Goodacre R., Griffin J.L., Hardy N.W., Kaddurah-Daouk R., Kristal B.S., Lindon J., Mendes P., Morrison N. (2007). The metabolomics standards initiative (MSI). Nat. Biotechnol..

